# Comment on “Pulmonary Function Tests in Hypertensive Patients Attending Zewditu Memorial Hospital, Addis Ababa, Ethiopia”

**DOI:** 10.1155/2019/5167018

**Published:** 2019-04-04

**Authors:** Yusuf Ziya Şener, Vedat Hekimsoy, Metin Okşul

**Affiliations:** Hacettepe University Faculty of Medicine, Cardiology Department, Sıhhiye, Ankara, Turkey

We have read with great interest the article published by Birhan et al., which was about pulmonary function tests in patients with hypertension [1]. It is worth noting that patients using beta blockers were excluded from the study and some parameters of the pulmonary function tests were significantly lower in patients with hypertension than in healthy counterparts.

It is well known that antihypertensive medications have certain effects on pulmonary functions. Beta blocking agents cause bronchoconstriction, exacerbating attacks in patients with asthma and COPD. Dry cough is a hallmark complication of treatment with ACE inhibitors. ACE degrades biologically active peptides, including bradykinin, and substance P, and ACE inhibitors lead to accumulation of these peptides. Therefore, ACE inhibitors may cause bronchoconstriction. It is also demonstrated that furosemide has preventive effects on bronchoconstriction. Hydrochlorothiazide can cause interstitial pneumonia in some patients [2]. An association between hypoxia and hypertension is documented, especially in patients with sleep apnea and obesity-hypoventilation syndrome [3]. So restrictive pattern-impaired pulmonary functions may be the cause of increased blood pressure, rather than consequence of hypertension.

To conclude, we think that it would be better if the patients' antihypertensive medications and blood oxygen saturation levels were evaluated.
